# A Case of Takayasu's Arteritis Presenting With Acute Middle Cerebral Artery Stroke Managed With Aortic-Common Carotid Artery (CCA) Bypass Surgery

**DOI:** 10.7759/cureus.48166

**Published:** 2023-11-02

**Authors:** Ghalib Nashaat El Hunjul, Juliana Cazzaniga, Jhon Navarro Gonzalez, Jonathan Quinonez, Samir Ruxmohan, Abrahim Fahs

**Affiliations:** 1 Internal Medicine, Soba University Hospital, Khartoum, SDN; 2 Neurology, Florida International University, Herbert Wertheim College of Medicine, Miami, USA; 3 Neurology, University of Zulia, Maracaibo, VEN; 4 Osteopathic Medicine/Neurology, Larkin Community Hospital Palm Springs Campus, Hialeah, USA; 5 Addiction Medicine, Brandon Regional Hospital, Brandon, USA; 6 Neurocritical Care, University of Texas (UT) Southwestern Medical Center, Dallas, USA

**Keywords:** immunosuppressive therapy, neurocritical care, neurology, syncope, takayasu's arteritis

## Abstract

Takayasu's arteritis (TA) is a rare inflammatory disorder that affects large arteries, particularly the aorta and its main branches. TA is also known as a pulseless disease because it diminishes blood flow to the limbs and organs. The patient was a 17-year-old female whose prior medical history included a diagnosis of TA. She had been experiencing multiple syncopal episodes up to three times daily, lasting 10 seconds each. She was being managed outpatient with immunologic therapy and warfarin. She initially presented to a children's hospital with abdominal pain and an asymmetrical smile and was found to have a ruptured ovarian cyst. This case demonstrated that life-threatening complications of TA can occur as a result of otherwise unrelated and common circumstances. The patient was managed medically and then proceeded to surgery. The case further highlights the multidisciplinary team approach between medical and surgical specialties and weighing the risks and benefits of complications for the patient's long-term care. Early diagnosis and prompt initiation of appropriate therapy are essential for better outcomes. Clinicians should be aware of the nonspecific symptoms of TA and consider it in the differential diagnosis of young patients presenting with systemic symptoms and arterial insufficiency. The initial presentation of middle cerebral artery stroke in young women has been documented in prior literature, but most published cases present the medical management of the disease. Our patient's case was unique because medical management was insufficient, with surgical management pursued due to persistent symptomatic hypotension. The inciting event of this case, an ovarian rupture with retroperitoneal hemorrhage, represents a unique burden to watershed infarctions in this patient group. Further research is needed to understand the pathogenesis of TA better and to develop more effective treatment strategies for this challenging disease.

## Introduction

Takayasu's arteritis (TA) is a rare inflammatory disorder affecting large vessels, primarily the aorta and its main branches [1]. TA is also known as a pulseless disease because it diminishes blood flow to the limbs and organs. First characterized in 1905, TA is more prevalent in females and mainly affects patients younger than 50 [1]. Since then, the disease has been reported worldwide, with the highest prevalence in Asia, most notably in Japan [2]. TA causes immune-mediated inflammation, leading to transmural fibrosis of large and medium arteries, which obstructs and weakens the arterial walls. These changes reduce blood flow to vital organs such as the heart, brain, and kidneys [2,3]. Clinical features of TA depend on the severity of vascular involvement [2]. Patients may suffer from limb claudication, absent pulse, visual impairment, and other cardiovascular, neurological, and gastrointestinal complications [3,4]. Moreover, TA diagnosis can be challenging and requires high clinical suspicion due to the absence of clear diagnostic criteria and the presence of nonspecific symptoms [5].

Imaging modalities such as angiography, computed tomography angiography (CTA), magnetic resonance angiography (MRA), ultrasonography, and positron emission tomography (PET) can provide crucial information to diagnose the disease in its early stages and monitor disease progression [5,6]. Managing TA requires a multidisciplinary approach, which may include immunosuppressive and anti-inflammatory medications and, in severe cases, surgery [7]. We present the case of a female patient (age 17) with known TA and a history of multiple syncopal episodes.

## Case presentation

The patient was a 17-year-old female whose medical history included a diagnosis of TA with the patient's medical history, physical examination, blood tests, and imaging studies. She had been experiencing multiple syncopal episodes up to three times daily, lasting 10 seconds each. She was being managed as an outpatient with immunologic therapy and warfarin. She initially presented to a children's hospital with abdominal pain and an asymmetrical smile with left-sided facial droop and was found to have a ruptured ovarian cyst. CT pelvis revealed bilateral enlarged irregular heterogeneously enhancing masses, suggesting abnormal-appearing uterus and ovaries with intraperitoneal hemorrhage, likely worsened by chronic warfarin use.

The patient's general examination revealed that she was alert and oriented (AAOx3), with intact memory and an appropriate fund of knowledge. In the cardiovascular system, there were no abnormal findings, with regular rate and rhythm, normal dorsalis pedis pulse and posterior tibialis bilaterally (+4), but decreased intensity on the radial artery bilaterally (+1). In the chest, there were no signs of respiratory distress, and the patient was breathing comfortably on room air. Abdominal examination showed softness and non-tenderness to palpation. Moving to the neurologic assessment, the patient's mental status, language, and speech functions were intact, including naming, repetition, and the ability to follow three-step commands. The patient was awake, alert, and oriented to self, others, place, time, and situation. She had fluent speech and all visual fields were intact. Furthermore, the patient had a negative pronator drift. Cranial nerve evaluation revealed normal findings in various cranial nerves, including normal pupillary reactions and extraocular movements. Motor examination indicated symmetrical and full muscle strength bilaterally in the upper and lower extremities (5/5). Reflexes were graded 2/4 throughout with a flexor plantar response. Sensory examination was normal, except decreased sensation to light touch in bilateral lateral eyebrow regions (+1). There were no signs of neglect or extinction. Romberg's test was negative, coordination was normal, and the patient's gait was unremarkable, with the ability to tip-toe and heel-walk. 

**Figure 1:**
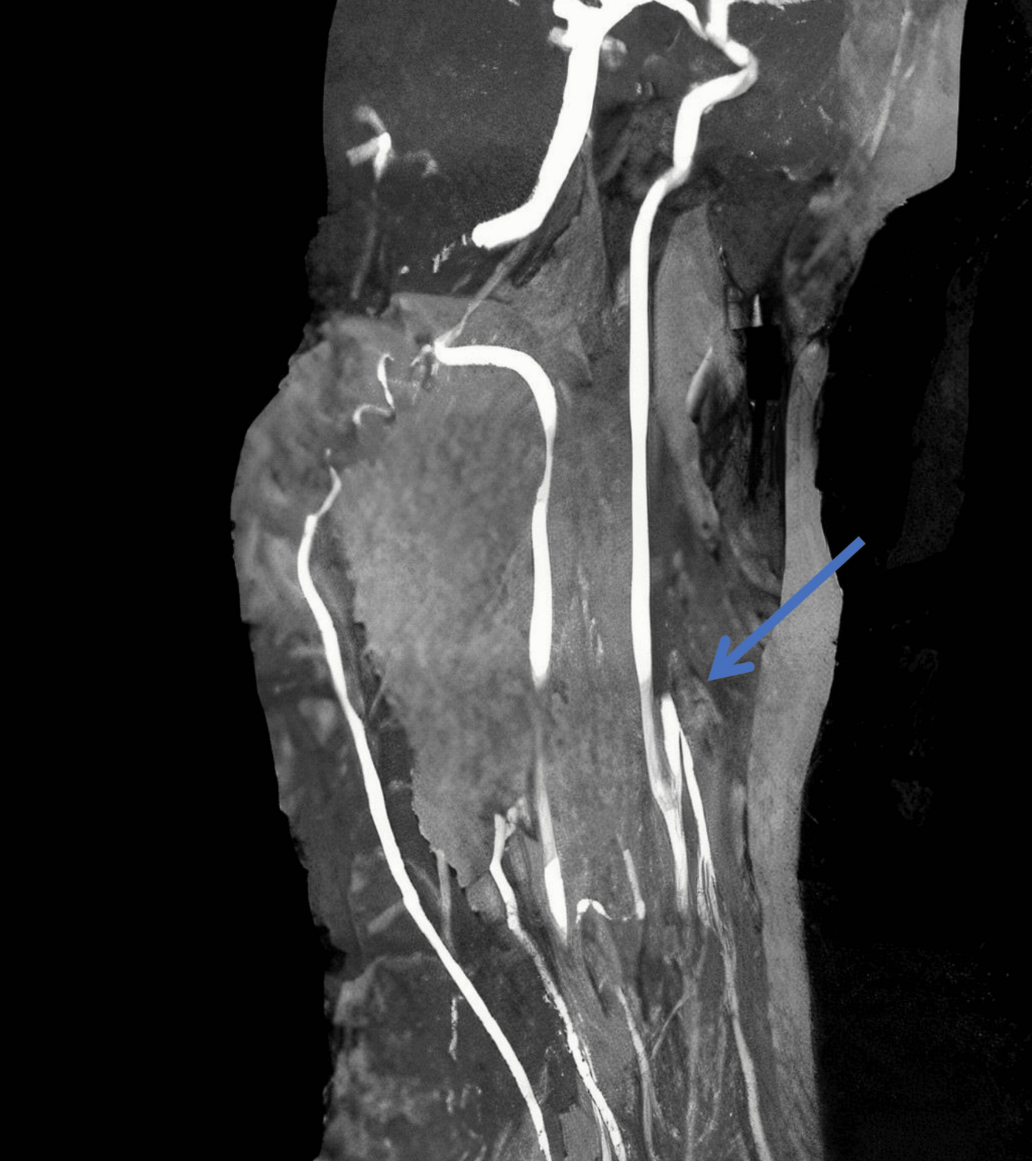
MRI of the neck with contrast shows the presence of severe bilateral carotid arterial narrowing shown with the blue arrow

**Figure 2:**
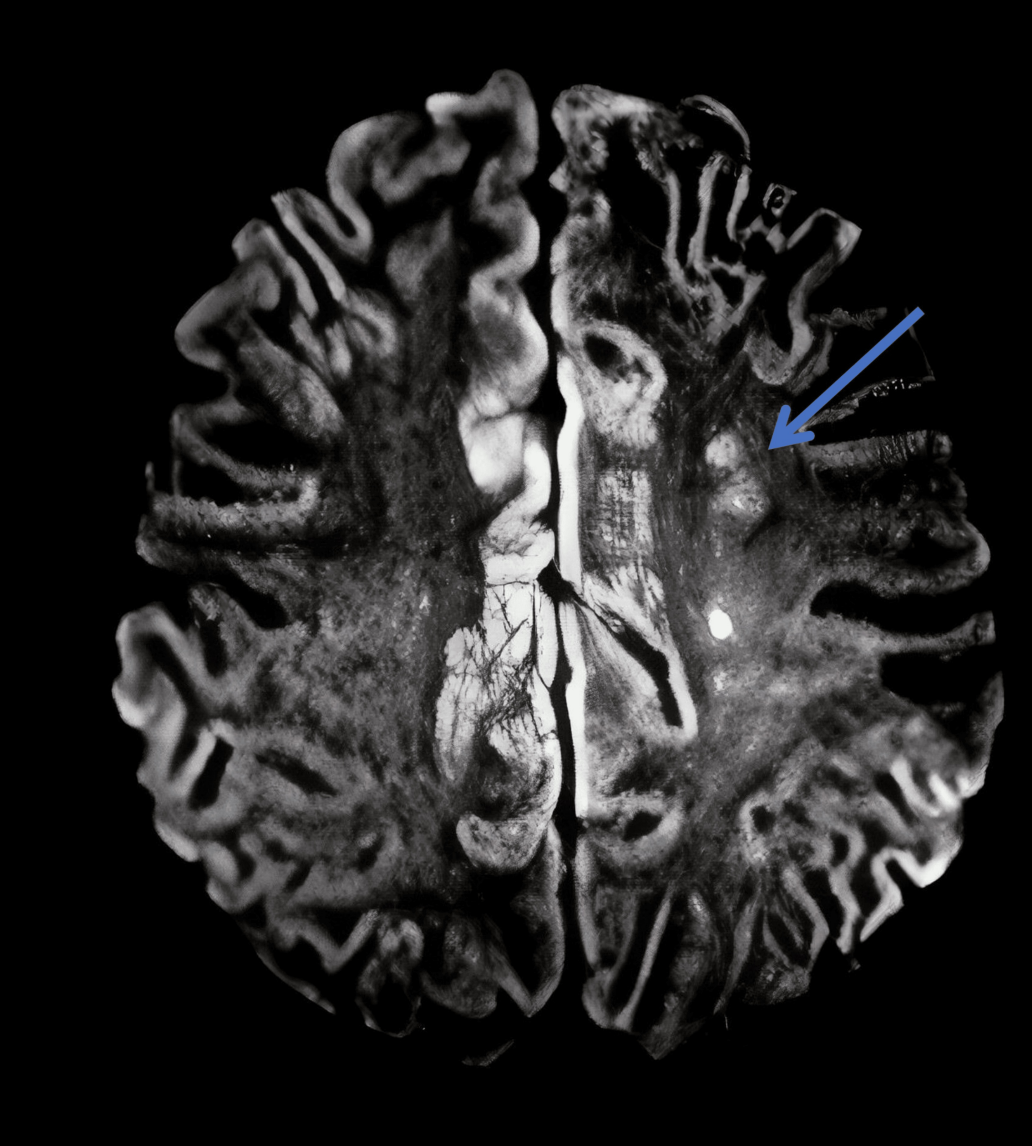
MRI scans of the head with contrast shows the presence of white matter infarcts of the left frontal and parietal regions shown with the blue arrow

Upper extremity blood pressures were undetectable, and lower extremity blood pressures were in the range of 160-220 systolic and 80-120 diastolic. She had a left middle cerebral artery infarct on MRI with diffuse irregularity to the left M1 segment. Isradipine was tried on pediatric ICU hospital day 2 to decrease lower extremity hypertension. Imaging revealed bilaterally narrowing of carotid arteries (Figure 1) and white matter infarcts of the left frontal and parietal regions from the watershed area of the anterior cerebral artery/middle cerebral artery (Figure 2). The physical exam was redone and demonstrated left hip weakness (3/5). Since the patient had prior intraperitoneal hemorrhage, thrombolytic therapy was not done due to increased risk of bleeding. Laboratory findings are shown in Table 1. Furthermore, the patient's creatine kinase total was 11 mg/dL (low), glucose was 116 mg/dL (high), WBC was 18.03 ^109/L (high), RBC was 5.43 K/uL (high), and Hb was 18 g/dL (high). Other lab results are shown in Table 1. 

**Table 1:** The patient's laboratory values during her hospital course CK: creatine kinase; hs-CRP: high-sensitivity C-reactive protein; hs-TnI: high-sensitivity troponin I; Na: sodium; K: potassium; Cl: chlorine; CO_2_: carbon dioxide; BUN/CREAT: blood urea nitrogen/creatinine; Ca: calcium; A/G: albumin/globulin; Mg: magnesium; P: phosphorus; GI: gastrointestinal; Alk Phos: alkaline phosphatase; ALT: alanine transaminase; AST: aspartate aminotransferase; BT: bacterial translocation; Vit B12: vitamin B12; LDL: low-density lipoprotein; HDL: high-density lipoprotein; TG: triglyceride; Chol: cholesterol; T4: thyroxine; TSH: thyroid-stimulating hormone; CBC: complete blood count; WBC: white blood cell; RBC: red blood cell; Hb: hemoglobin; MCV: mean corpuscular volume; MCH: mean corpuscular hemoglobin; MCHC: mean corpuscular hemoglobin concentration; RDW: red cell distribution width; MPV: mean platelet volume; NRBC: nucleated red blood cell; ABS: absolute; NEUTROS: neutrophils in white blood cells; PCT: procalcitonin; Gran: granulocyte; LYMPHS: lymphocytes; MONOS: monocytes; EOS: eosinophils; BASOS: basophils; PT: prothrombin time; INR: international normalized ratio; PTT: partial thromboplastin time Up arrows (↑) represent a high value; down arrows (↓) represent a low value

Cardiac profile (units)	—	Diabetes (units)	
CK total (U/L)	11 ↓	Glucose (mg/dL)	116↑
hs-CRP (mg/L)	<0.2	A1C (mg/dL)	5.1
hs-TnI (ng/mL)	<5.0		
		Thyroid (units)	
Chemical profile (units)		Free T4 (ng/dL)	0.93
Na (mEq/L)	138	TSH (ng/dL)	0.24↓
K (mEq/L)	4.3		
Cl (mEq/L)	102	CBC (units)	
CO_2_ (ppm)	22	WBC (K/uL)	18.03↑
Anion gap (mEq/L)	14	RBC (K/uL)	5.43↑
Glucose (mg/dL)	116 ↑	Hb (g/dL)	18.0↑
Creatinine (mg/dL)	0.49 ↓	Hematocrit (%)	50.0↑
BUN/CREAT (mg/dL)	28.6↑	MCV (pg/cell)	93.4
Ca (mEq/L)	10.3↑	MCH (pg/cell)	33.1↑
Ca (albumin adjusted)	9.7	MCHC (pg/cell)	35.5
Protein T (g/L)	8.1	RDW (pg/cell)	13.6
Albumin (g/L)	4.8↑	Platelets (K/uL)	270
Globulin (g/L)	3.3	MPV (pg/cell)	10.1
A/G ratio	1.5	NRBC (K/uL)	0.0
Mg (mEq/L)	2.4	NRBC ABS (K/uL)	0.00
P (mEq/L)	4.4		
		Differential (units)	
GI liver profile (units)		NEUTROS PCT (K/uL)	93.6
Alk Phos (U/L)	64	Immature Gran %	0.7
ALT (U/L)	40↑	LYMPHS PCT %	4.3
AST (U/L)	31	MONOS PCT %	1.1
BT (mg/dL)	1.0	EOS PCT %	0.0
		BASOS PCT %	0.3
Iron/anemia profile (units)		NEUTROS ABS (K/uL)	16.89↑
Vit B12 (mcg)	619	Immature Gran ABS (K/uL)	0.12↑
		LYMPHS ABS (K/uL)	0.77↓
Lipid profile (units)		MONOS ABS (K/uL)	0.19
Chol (mg/dL)	179		
LDL (mg/dL)	92.4		
HDL (mg/dL)	64	Coagulation (units)	
TG (mg/dL)	113	PT (s)	9.8
Non-HDL Chol (mg/dL)	115	INR (s)	0.9
Chol/HDL ratio	2.8	PTT (s)	25.7

Further workup of CTA head and neck with contrast revealed luminal narrowing/irregularities (left worse than right), left proximal subclavian artery, and left vertebral artery. The left common carotid artery (CCA) was severely narrowed with irregular circumferential wall thickening and little contrast in the lumen. The left internal and external carotid arteries were patent but small in caliber. Carotid duplex revealed >70% stenosis of the proximal right CCA and narrowing of the proximal and mid-left CCA with non-quantifiable, minimally pulsatile velocity. A transthoracic echocardiogram showed pulmonary hypertension.

From a rheumatologic perspective, the patient had normal serum inflammatory markers. Initial TA management included 60 mg methylprednisolone and tocilizumab. On day 4 of admission, she received cyclophosphamide, and aspirin 81 mg was restarted due to a stroke. She continued to have symptomatic hypotension throughout her hospitalization, was treated with midodrine, and did not have any syncopal episodes during hospitalization. The medical team was focused on maintaining mean arterial pressure (MAP) above 65 and systolic blood pressure (SBP) below 180 mm Hg unless she developed signs of end-organ injury.

On day 17, she underwent median sternotomy and aortic right axillary bypass with a 6-mm-ringed GORE-TEX tube graft (W. L. Gore & Associates, Inc., Flagstaff, Arizona, United States) and aorta right CCA bypass with a 6-mm-ringed vascular tube graft. Imaging showed a patent graft and development of multifocal acute infarcts in the left frontoparietal lobes, right temporal lobe, left occipital lobe, and left cerebellar hemisphere, with an increased size of the right caudate acute/subacute infarct and an additional tiny subacute infarct in the left frontal horn periventricular white matter. At discharge on day 22, the patient required extensive physical and occupational therapy. The patient was discharged to inpatient rehab after hospitalization.

One month later, she could ambulate easily and stopped having syncopal episodes. She continued to have left hip weakness and was slightly weaker on the left side (3/5). Her outpatient rheumatologic medications included daily corticosteroids, methotrexate, and tocilizumab. She was also receiving cyclophosphamide infusions. As a result of follow-up visits and noted improvement, her steroids were reduced. She continued to be anticoagulated with aspirin and clopidogrel. Her one-month follow-up echocardiogram appeared normal, and her pulmonary hypertension responded to sildenafil.

## Discussion

We present the case of a 17-year-old female whose medical history included a diagnosis of TA in September 2022. She had been experiencing multiple syncopal episodes up to three times daily, lasting 10 seconds each. In the context of this case, the presence of TA introduced several potential factors contributing to the occurrence of multiple syncopal episodes, for example, the stenosis or narrowing of the carotid arteries could have significantly impeded the blood flow to the brain, particularly within the territory of the middle cerebral artery. This constriction in blood flow resulted in cerebral hypoperfusion and the potential to precipitate syncope. Furthermore, TA predisposed her to cerebrovascular events, with strokes being a noteworthy concern. Syncopal episodes in this case may be associated with such cerebrovascular events, possibly initiated by the formation of small embolic events or thrombi within the cerebral arteries. These events can lead to transient or even persistent neurological deficits, further increasing the risk of syncope. The disease's influence on arterial stiffness and arterial supply compromised gave rise to hemodynamic instability. Abrupt changes in blood pressure or cardiac output, triggered by the underlying vascular abnormalities, served as potential provocateurs of syncope in this complex medical scenario of this patient. Additionally, she was being managed outpatient with immunologic therapy and warfarin. The initial presentation of MCA stroke in young women has been documented in prior literature, but most case reports present the medical management of the disease [8]. Our patient's case was unique because medical management was insufficient, with surgical management pursued due to persistent symptomatic hypotension. The inciting event of this case, an ovarian rupture with retroperitoneal hemorrhage, represents a unique susceptibility to watershed infarctions in this patient group. Our patient had a normal erythrocyte sedimentation rate (ESR), which is common for TA patients [9]. However, ESR is typically considered to be a marker of active disease. Elevated ESR is associated with higher mortality rates and a sevenfold complication increase in surgical cases. Additionally, hyperlipidemia, hypertension, and higher steroid doses are associated with surgery-related vascular complications [10]. 

The medical management of TA includes immunosuppressive therapy, such as corticosteroids and immunosuppressive agents. In some prior cases, the initial presentation of TA was presented as a stroke in young Hispanic females [11]. Strokes are a lethal complication to an initial TA diagnosis in children and adults [12]. The primary treatment for TA is corticosteroids, and it has been shown that 50% of patients can achieve remission with steroid therapy alone [12]. For TA resistant to steroid treatment, cytotoxic therapy is frequently introduced. Cytotoxic medications such as methotrexate and tocilizumab have been evaluated for the treatment of TA, with the current practice being to choose based on the side effect profile [1,13]. Overall, steroids treat about 50% of patients, with methotrexate as an additional drug to treat the other 50% of patients [1]. In a prospective study, one-third of patients receiving glucocorticoids and cytotoxic therapy went into remission within an average time of 20 months [7]. In our case, the patient was severely symptomatic, and medical treatment alone was unsuitable for treating her symptoms and disease severity.

Typically, surgical options are required for greater than 70% stenosis of the carotid arteries, critical heart ischemia, hypertension with end-organ damage, and aortic regurgitation [12]. Patients with TA younger than 20 years old have a 3.5 higher odds ratio of having vascular surgery than older patients [14]. Our patient responded well to surgery and showed continued improvement with medical management at her follow-up visits. Bypass surgery quickly decreased the severity of her symptoms and allowed immunosuppressive therapy to serve as a maintenance treatment.

## Conclusions

TA is a rare but serious disease that can cause significant morbidity and mortality if left untreated. Early diagnosis and prompt initiation of appropriate therapy are essential for better outcomes. Clinicians should be aware of the nonspecific symptoms of TA and consider it in the differential diagnosis of young patients presenting with systemic symptoms and arterial insufficiency. Further research is needed to understand the pathogenesis of Takayasu's arteritis better and to develop more effective treatment strategies for this challenging disease. Additional insights into the MCA stroke in this case are essential. It is crucial to specify the precise location of the MCA infarct, as the clinical manifestations can vary significantly depending on the affected area of the brain. The neurological exam focused on motor weakness, sensory loss, language difficulties, or visual disturbances. Understanding the potential impact of the MCA stroke on the patient's daily life is equally important, as it enables a comprehensive assessment of any impairments in movement, communication, or cognition for the determination of surgical treatment and rehabilitation plan. 

The decision to proceed with surgery in this case was driven by a combination of crucial factors due to the severity of the vascular involvement, particularly the significant stenosis observed in the carotid arteries. Also, the presence of critical ischemia was a serious threat to vital organs and neurological function, making surgical intervention a vital step. Persistent symptomatic hypotension further underscored the need for surgery, as addressing this issue was essential to improve blood flow and overall patient well-being and prevent future syncopal episodes. Moreover, the choice for surgical intervention aimed not only at immediate relief but also at facilitating better long-term management, working in conjunction with the immunosuppressive therapy. Overall, the patient's quality of life was an important factor to consider with a primary focus on improving outcomes and providing relief from the severe symptoms associated with her diagnosis of TA.
