# Correction: A Case of Takayasu's Arteritis Presenting With Acute Middle Cerebral Artery Stroke Managed With Aortic-Common Carotid Artery (CCA) Bypass Surgery

**DOI:** 10.7759/cureus.c372

**Published:** 2025-10-31

**Authors:** Ghalib Nashaat El Hunjul, Juliana Cazzaniga, Jhon Navarro Gonzalez, Jonathan Quinonez, Samir Ruxmohan, Abrahim Fahs

**Affiliations:** 1 Internal Medicine, Soba University Hospital, Khartoum, SDN; 2 Neurology, Florida International University, Herbert Wertheim College of Medicine, Miami, USA; 3 Neurology, University of Zulia, Maracaibo, VEN; 4 Osteopathic Medicine/Neurology, Larkin Community Hospital Palm Springs Campus, Hialeah, USA; 5 Addiction Medicine, Brandon Regional Hospital, Brandon, USA; 6 Neurocritical Care, University of Texas (UT) Southwestern Medical Center, Dallas, USA

This article's ethics statement has been corrected to indicate that it does contain human subjects and informed consent was obtained prior to publication.

